# A Cross-Sectional Study on the Prevalence of Skipping Breakfast Among College-Going Students in a Private University

**DOI:** 10.7759/cureus.76700

**Published:** 2024-12-31

**Authors:** Lakshmi Nagalingam, J Princy Felicia, Aishwarya P M, Nithya V

**Affiliations:** 1 Community Medicine, Sree Balaji Medical College and Hospital, Chennai, IND; 2 Community Medicine, Madras Medical College, Chennai, IND

**Keywords:** bmi, breakfast skipping, eating habits, lifestyle, nutrition

## Abstract

Background

Breakfast is considered one of the important meals of the day as it repletes glucose supply to the brain and improves learning. Skipping breakfast has become more common among college students. It is prompting them to adopt unhealthy lifestyles which might affect their health in the long term. The present study aimed to assess the health effects of skipping breakfast among college students. The main objective of this study is to estimate the prevalence of breakfast-skipping patterns and the factors associated with skipping breakfast among college students.

Methods

This descriptive cross-sectional study involved 540 college students from four institutions (Karpaga Vinayaga Institute of Medical Sciences and Research Centre, Chengalpattu, Karpaga Vinayaga Institute of Dental Sciences, Chengalpattu, Karpaga Vinayaga Institute of Dental Sciences, Chengalpattu, and Karpaga Vinayaga College of Nursing, Chengalpattu) in Tamil Nadu. A Simple Random Sampling method was used to select the sample. Data were gathered using a pre-tested, semi-structured questionnaire, and ethical clearance was obtained from the institution. The data were analyzed with IBM SPSS Statistics for Windows, Version 21 (Released 2012; IBM Corp., Armonk, New York, United States), employing both descriptive and inferential statistics.

Results

The prevalence of breakfast skipping among college-going students was 368 (68.2%). The timing of breakfast was a strong predictor; those eating after 10 AM had an adjusted odds ratio of 3.98 (95% CI: 1.65 - 9.61). The use of snacks as meal replacements also showed a significant association, with an odds ratio of 2.90 (95% CI: 1.95 - 4.32). Spending pocket money on breakfast was linked to an increased likelihood of skipping, with an adjusted odds ratio of 1.251 (95% CI: 0.82 - 1.90).

Conclusion

This study shows that a higher proportion of college students skip their breakfast at least once a week. This study also showed that male gender, lower socioeconomic status, mealtime after 11 a.m., and snacking as a meal substitute more than four times a week are important risk factors for college students who were skipping breakfast. The results indicate that specific programs that encourage eating breakfast earlier and adopting healthy eating behavior may enhance student's general dietary habits.

## Introduction

Breakfast is often referred to be the most essential meal of the day, and in recent times, it has been linked to metabolic and cardiovascular risk factors, weight management, and cognitive function [1]. The proposed definition is “breakfast is the first meal of the day that breaks overnight fast and is normally consumed within two to three hours of awakening. It consists of food or drink from at least one food group and can be eaten anywhere [2]. Breakfast helps burn calories throughout the day by boosting metabolism. It was generally the first meal of the day and it should be taken before 10 a.m. of that day [3]. Meanwhile, there were no standards for the nutrients of adequate breakfast. It should generally provide 20-25% of the recommended daily intake. In addition to providing other essential nutrients for optimum health, its practical advantages include enhanced alertness, improved energy, and replenishing the glucose supply [4].

Youngsters who have breakfast daily typically consume fewer calories, as well as higher amounts of dietary fiber, calcium, iron, folate, and total carbohydrates [5]. In developed countries like the US and Europe, the prevalence of breakfast skipping varies from 10 to 30%. Breakfast eaters are less likely to be overweight compared to students who skip breakfast and eat snacks as their meal replacement [6]. Increased attendance and decreased tardiness are the two most potent and frequently mentioned ways that breakfast may increase learning outcomes [7]. Although daily breakfast offers many advantages, it is estimated that globally, 10 to 30% of children and adolescents regularly skip this meal. Among university students, about 64.3% skipped their breakfast in Africa, 51.2% in Turkey, and 55.2% in Pakistan, which on average comes to about 56.9% with a median age of 20 [8]. However, in India, the pattern of skipping breakfast can go high up to 66% in Tamil Nadu [9].

Breakfast intake is one of the behavioral and psychological changes that take place during the transition period from school life. When youngsters move away from home and attend college, where they must be self-sufficient, their eating habits tend to deteriorate and they start consuming fast food [10]. It is impossible to overestimate the influence breakfast has on the performance and health of young people, especially college students. This is because, in addition to its relationship to proper nutrition, breakfast improves young people's attention, focus, intellectual capacity, and eventually academic performance [11].

Skipping breakfast is defined as intentionally or unintentionally missing a meal at least once a week [8]. Health consequences such as migraine, overweight, type 2 diabetes, hypoglycemia, and hair loss are linked to skipping breakfast. Additionally, skipping breakfast is a modifiable factor associated with the risk of overweight and obesity, as well as unhealthy eating habits in children and adolescents [12]. A systematic review by Ofori-Asenso et al. found that people who had the behavior of skipping breakfast regularly were 21% more likely to experience cardiovascular diseases. The risk of all causes of death was 32% more likely to occur among people with skipping breakfast patterns [13]. So, the present study aimed to assess the health effects of skipping breakfast among college students with the objective of estimating the prevalence of breakfast-skipping patterns and the factors associated with skipping breakfast among college students in Chengalpattu.

## Materials and methods

A cross-sectional study was done among college students in Chengalpattu district during the period of three months from June 2024 to August 2024.

The sample size was estimated using the formula, 4pq/d^2^, (p=expected proportion of estimate in population; q=1-p; d=absolute precision of estimate to the true value of population parameter; 4 is the approximated value of the square of z(1-α/2) i.e. 1.96, which is the standard normal distribution value corresponding to a significance level of α at 0.05). According to Joy, the prevalence of skipping breakfast among college students from East West University was found to be 31%, and the sample size was calculated to be 534 (p=31%, q=69%, d=absolute error of 4%), which was rounded off to 540 [14].

The university chosen in the district had various streams of education namely, Medical, Dental, Engineering, and Nursing. The minimum sample size required was 540. In order to arrive at the required sample size, 135 students were chosen from each stream of education. The approximate sampling frame in each stream of education was 600 (Medical), 400 (Engineering), 280 (Nursing), and 386 (Dental). From each stream of education, 135 students were chosen randomly by using a random number generator to get the required sample size. The study protocol was presented to the Institutional Scientific Committee and Ethical Committee to adhere to ethical guidelines and the study was approved by them. The students were selected using a random number generator and those who agreed to participate in the study were included. The individuals who were absent on the day of the study and individuals with chronic health problems like diabetes were excluded and the next random number was chosen.

A pre-tested semi-structured questionnaire was used to collect the data. The questionnaire was designed based on the literature review [15,16]. A pilot study was done among 30 participants and modification of content was done in the questionnaire based on expert opinion. The participants from the pilot study were excluded from the analysis.

The definition for skipping breakfast is given as “the act of not taking any food or beverage between 05.00 and 10.00 hours is known as skipping breakfast, which can be done occasionally, infrequently, or never". In our study, we included skipping breakfast for the past week using the recall method [17].

The data were entered and analyzed using Microsoft Excel 2019 (Microsoft Corporation, Redmond, USA) and IBM SPSS Statistics for Windows, Version 21 (Released 2012; IBM Corp., Armonk, New York, United States), respectively. The categorical variables were expressed as proportions. Association of demographic variables, behavioral habits, and skipping of breakfast was tested using a chi-square test between the predictor and outcome variables. The binary logistic regression analysis was performed to find the independent predictors of skipping breakfast.

## Results

This study was conducted among 540 students from four different colleges to determine the prevalence of breakfast-skipping patterns. The socio-demographic characteristics of the study participants are shown in Table 1. The mean age of college students was 19.61±1.404 years ranging from 17 to 25 years. Out of 540 study participants, 252 (46.7%) were men, and 288 (53.3%) were women. The majority of the students were studying in their first and second year of college and most of them were staying with either family or friends. The majority of the students belonged to Class II (upper-middle-class) socio-economic status classification followed by Class III (middle class), according to the modified B.G Prasad Socio-Economic Status Scale, 2024 [18].

**Table 1:** Socio-demographic characteristics of the study participants (n=540)

S No	Variables		Frequency (n)	Proportion(%)
1	Gender	Male	252	46.7%
		Female	288	53.3%
2	Year of the study	First-year	154	28.5%
		Second year	217	40.2%
		Third year	88	16.3%
		Fourth-year	52	9.6%
		Intern	29	5.4%
3	Residential status	Hosteller	92	17.3%
		Family	223	41.3%
		Relatives	7	1.3%
		Friends	218	40.4%
4	Socio-economic status	Class I	115	21.3%
		Class II	212	39.3%
		Class III	144	26.7%
		Class IV	69	12.8%

**Figure 1:**
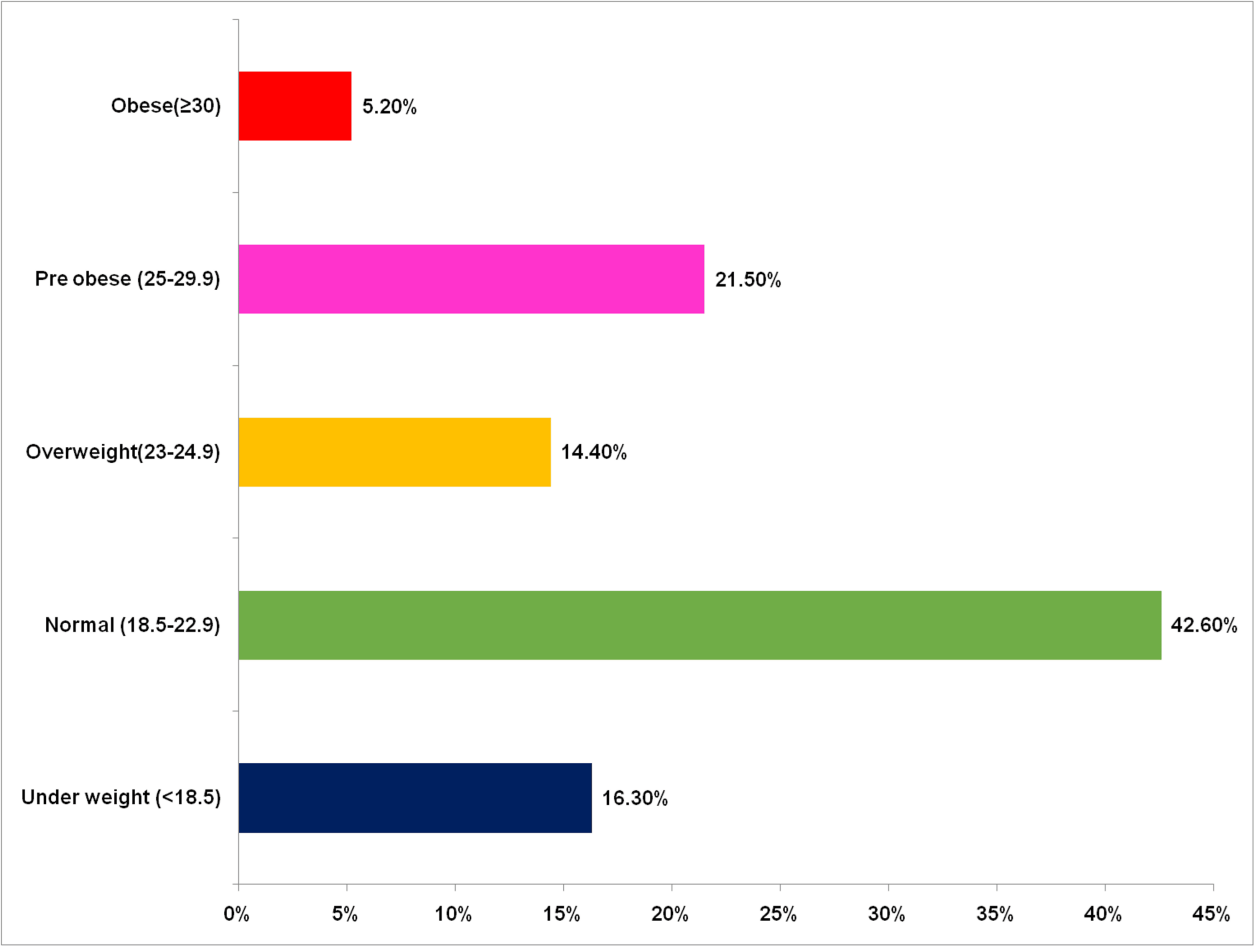
BMI classification based on Asian Indian classification among study subjects (n= 540)

Figure 1 shows the body mass index (BMI) classification based on Asian Indian classification among study subjects. According to the revised guidelines, BMI of <18.5 kg/m^2^ was considered underweight, 18.5-24.9 kg/m^2^ was considered normal, 23.0 - 24.9 kg/m^2^ was considered overweight, BMI of 25-29.9 kg/m^2^ as pre-obese, and BMI of >30 kg/m^2 ^as obesity. Although half of the study subjects were considered normal as per classification, 28 (5.2%) of them were obese and 88 (16.3%) were underweight.

**Figure 2:**
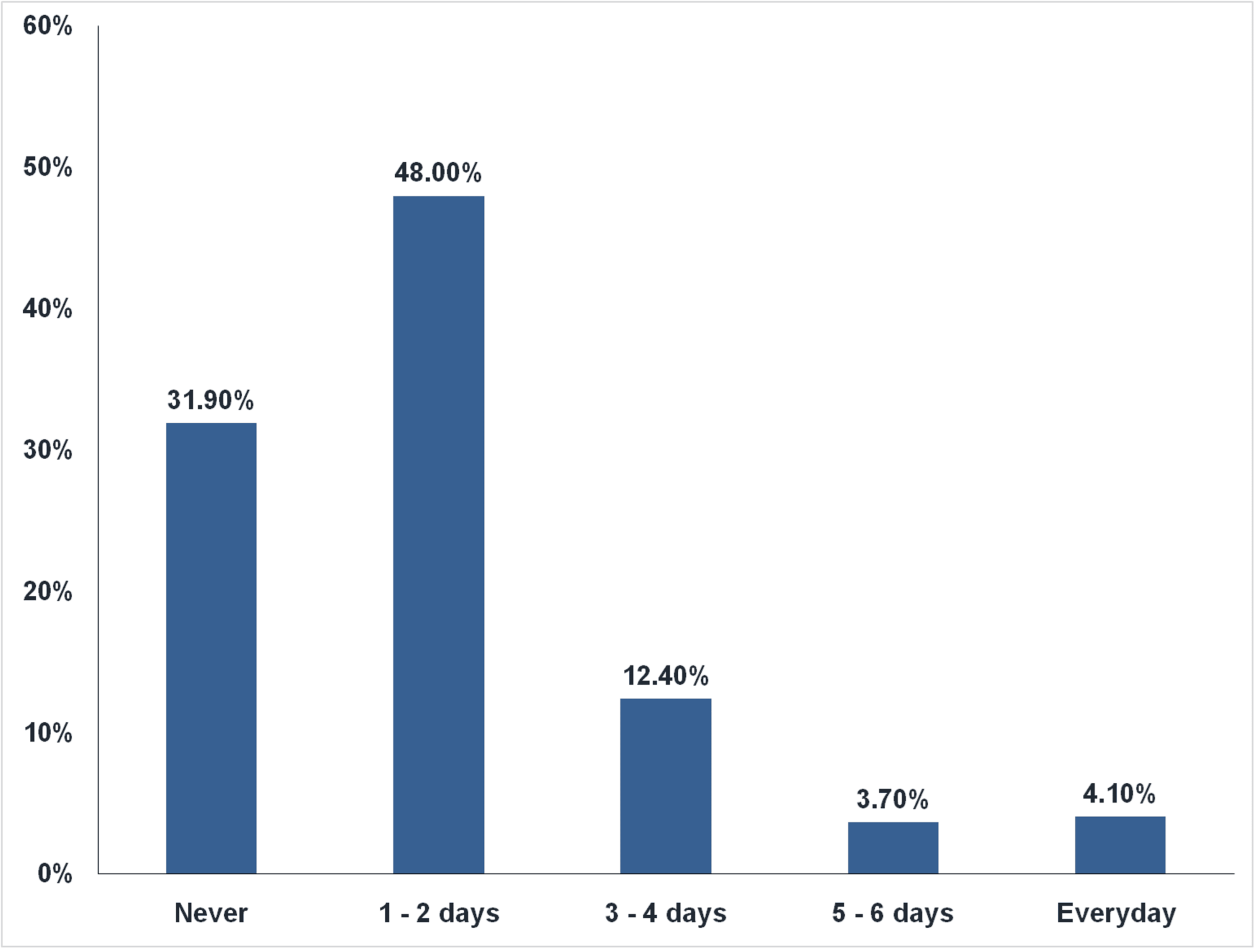
Frequency of skipping breakfast (n=540)

Figure 2 shows the frequency of skipping breakfast among study subjects. The study examined how often participants skip breakfast a week, revealing that 259 respondents (48%) miss breakfast 1 to 2 days per week, while 67 respondents (12.4%) and 20 respondents skip it for 3 - 4 days and 5 - 6 days respectively. In contrast, 172 respondents (31.9%) said they never miss breakfast and 22 respondents (4.1%) missed it daily. The prevalence of breakfast skipping among college-going students was 68.2%.

Table 2 describes the factors associated with breakfast. Among the study participants, 504 (93.3%) participants reported consuming traditional breakfast items like Pongal, idly, dosa, bread, or eggs. A small proportion of participants, 26 (4.8%) and 10 (1.8%), included milk and milk products or fruits and sprouts as their breakfast choices, respectively. A significant proportion of participants, 351 (65.1%) reported substituting breakfast with snacks, suggesting a common practice of opting for convenience over a traditional meal. Notably, only 189 (34.9%) reported never replacing breakfast, highlighting the prevalence of this behavior. The majority of participants, 246 (45.6%) consumed breakfast between 8 am and 10 am, while a nearly equal proportion 233 (43.1%) had breakfast before 8 am. A small fraction 61 (11.3%) had breakfast after 9 am, indicating that a significant portion of the population has breakfast later in the morning. The most common duration between dinner and next meal reported was 10 - 12 hours 177 (32.8%), suggesting that many participants maintain a moderate overnight fasting period. A total of 75 (13.97%) of respondents reported durations of 12 hours or more, indicating a tendency towards longer overnight fasting.

**Table 2:** Factors associated with breakfast (n=540)

S.No	Variables		Frequency (n)	Proportion (%)
1.	Type of meal	Idly/Dosa/Pongal/Bread/egg	504	93.3%
		Milk and Milk Products	26	4.8%
		Fruits & Sprouts	10	1.8%
2.	Snacks as a meal replacement	Once per week	191	35.4%
		2 – 3 times per week	108	20%
		At least 4 times per week	29	5.4%
		All day per week	23	4.3%
		Never	189	35%
3.	Timing of breakfast	Before 8 am	233	43.1%
		8 – 10 am	246	45.6%
		10 – 11 am	42	7.8%
		After 11 am	19	3.5%
4.	Minimum duration between dinner and next meal	6 – 8 hours	136	25.2%
		8 – 10 hours	152	28.1%
		10 – 12 hours	177	32.8%
		> 12 hours	75	13.9%

**Figure 3:**
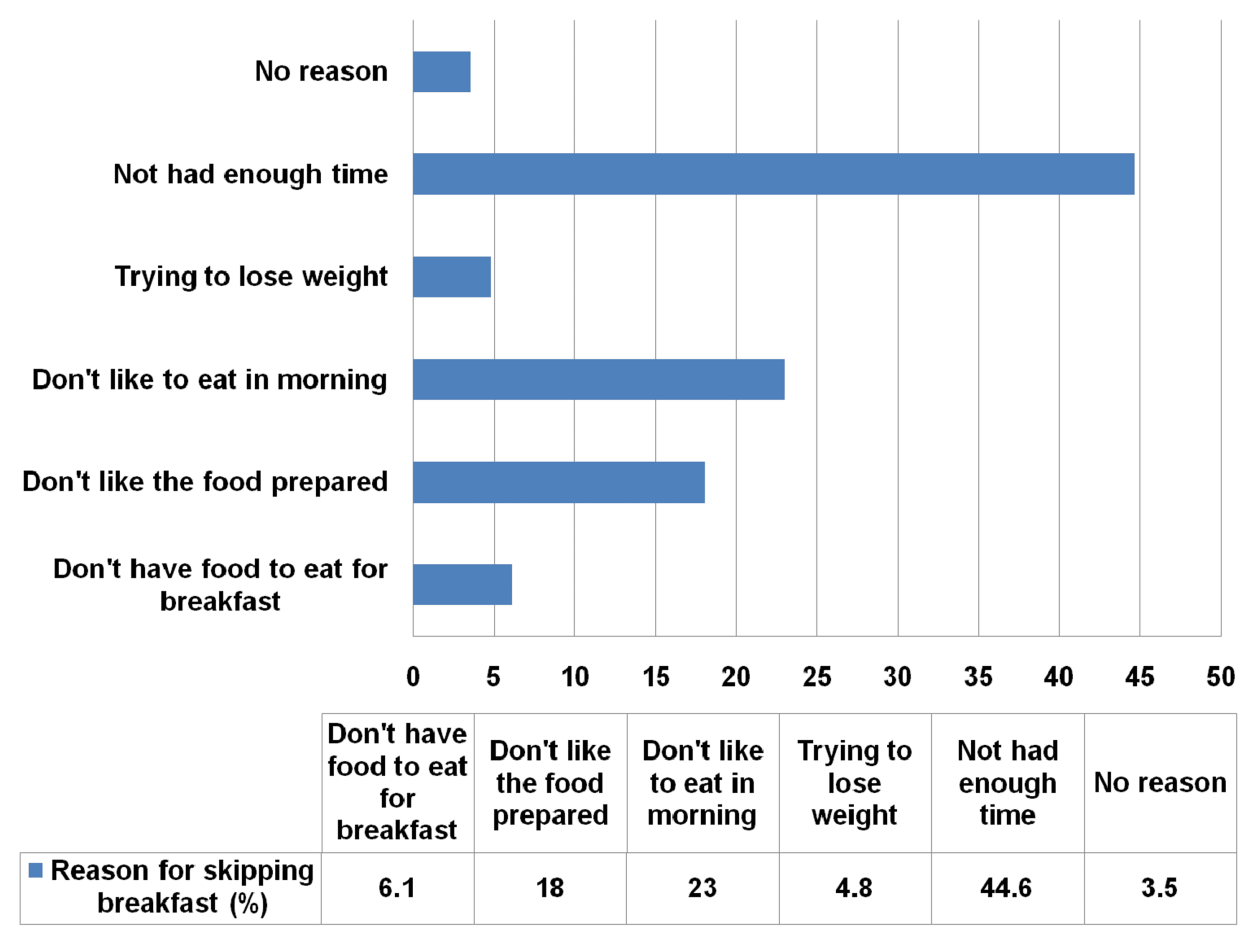
Reasons for skipping breakfast among study participants (n=540)

Figure 3 shows the reasons for skipping breakfast among study participants. A small proportion, 19 (3.9%), reported not having available foods, while nearly one-fifth 97 (18%) showed their dissatisfaction with available options for breakfast recipes. Additionally, 124 (23 %) informed that they don’t like to eat breakfast, and 26 (4.8%) of them mentioned that they were trying to lose weight, which influenced their decision to skip breakfast. Among the study participants, 33 (6.1%) did not have any food left to eat. However, most of the study participants 241 (44.6%) cited they skip breakfast because they get up late, indicating time management issues as a primary factor.

Table 3 shows the association between predictor variables and breakfast skipping. Gender and year of study emerged as significant factors associated with breakfast skipping, while socio-economic status showed a strong association with breakfast skipping. The residential status of the study participants and body mass index were found to be statistically insignificant with breakfast skipping. The male gender, the second year of the study, and Class III and IV show significant association with breakfast skipping.

**Table 3:** Association of demographic variables with breakfast skipping (n = 540) *p<0.05: Statistically significant at 95% CI.

S.No	Variables		Breakfast Skipping: Yes	Breakfast Skipping: No	Chi-square value	p-value
1.	Gender	Male	183 (72.6%)	69 (27.4%)	4.35	0.036*
		Female	185(64.2%)	103(35.7%)		
2.	Year of study	First-year	84(54.5%)	70(45.4%)	19.00	0.001*
		Second year	165(76.04%)	52(23.9%)		
		Third year	60(68.1%)	28(31.8%)		
		Fourth-year	38(73.08%)	14(26.9%)		
		Intern	21(72.4%)	8(27.5%)		
3.	Residential status	Hosteller	59 (64.1%)	33 (35.8%)	2.17	0.536
		Family	150(67.3%)	73(32.7%)		
		Relatives	6 (85.7%)	1 (14.2%)		
		Friends	153(70.1%)	65(70.1%)		
4.	BMI (Asian)	Underweight (<18.5)	63(71.5%)	25(28.4%)	2.23	0.692
		Normal (18.5-22.9)	161(70%)	69(30%)		
		Overweight (23-24.9)	49(62.8%)	29(37.1%)		
		Pre-obese (25-29.9)	76(65.5%)	40(34.4%)		
		Obese(≥30)	19(67.8%)	9(32.1%)		
5.	Socio-economic status	Class I	80(69.5%)	35(30.4%)	17.34	0.0006*
		Class II	124(58.4%)	88(41.5%)		
		Class III	113(78.4%)	31(21.5%)		
		Class IV	51(73.9%)	18(26.9%)		

Table 4 shows the association of variables with breakfast skipping. A significant association is seen between the minimum time between dinner and the next meal. Out of the 75 students (13.8%) who waited more than 12 hours, 60(80%) of them skipped their breakfast compared to 93 (68.4%) of those who waited 6-8 hours. The p-value is 0.009, which indicates statistical significance. Regarding breakfast timing, a very significant p-value of 0.001 indicates that 18(94.7%) of participants who eat after 11 a.m. completely skip their breakfast, although 181(73.6%) of those who eat between 8 a.m. to 10 a.m. and 36(85.7%) of those who eat between 10 a.m. to 11 a.m. showed a significantly lesser habit of skipping breakfast. Furthermore, there is a strong association between the frequency of snacking and skipping breakfast; 27(93.1%) of those who replace meals at least four times a week skip breakfast, and the p-value in this case is 0.001. On the other hand, just 97(51.3%) of people who never substitute snacks for meals skip breakfast. Finally, when looking at how much pocket money is spent on breakfast, participants who spend it "sometimes" report skipping breakfast at a greater rate of 108(75%), while those who "never" spend it report skipping it at a lower rate of 99(59.6%). This difference is statistically significant, with a p-value of 0.024. 

**Table 4:** Association of behavioral factors with breakfast skipping (n = 540) *p<0.05: Statistically significant at 95% CI.

S.No	Variables		Breakfast Skipping: Yes	Breakfast Skipping: No	Chi-square value	p-value
1.	Minimum duration between dinner and next meal	6 – 8 hours	93 (68.4%)	43 (31.6%)	11.3	0.01016*
		8 – 10 hours	109(71.7%)	43 (28.3%)		
		10 – 12 hours	106 (59.9%)	71 (40.1%)		
		≥ 12 hours	60 (80%)	15 (20%)		
2.	Timing of breakfast	Before 8 am	132 (56.7%)	101 (43.3%)	29.5	0.00001*
		8 – 10 am	181 (73.6%)	65 (26.4%)		
		10 – 11 am	36 (85.7%)	6 (14.3%)		
		After 11 am	18 (94.7%)	1(5.3%)		
3.	Spending snacks as a meal replacement	Once per week	131 (68.6%)	60 (31.4%)	53.6	0.00001*
		2 – 3 times per week	95 (88%)	13 (12%)		
		At least 4 times per week	27 (93.1%)	2 (6.9%)		
		All day per week	18 (78.3%)	5 (21.7%)		
		Never	97 (51.3%)	92 (48.7%)		
4.	Spending pocket money for breakfast	Very often	31 (66%)	16 (34%)	9.4	0.024*
		Sometimes	108 (75%)	36 (25%)		
		Rarely	130 (71%)	53 (29%)		
		Never	99 (59.6%)	67 (40.4%)		

The unadjusted and adjusted odds ratios (AOR) for the participants' risk variables for skipping breakfast were calculated. Men are more likely than women to skip breakfast, as seen by the AOR of 1.44 (95% CI: 1.00 - 2.08). After adjusting for other factors, the odds ratio becomes 1.27 (95% CI: 0.86 - 1.87), suggesting a reduced likelihood, with a significant p-value of 0.049. For participants who wait more than 10 hours between dinner and breakfast, the unadjusted odds ratio is 0.80 (95% CI: 0.55 - 1.15), and the AOR is 1.24 (95% CI: 0.86 - 1.78), with a p-value of 0.240, indicating no significant association. There is a substantial correlation between skipping breakfast and having a considerably higher unadjusted odds ratio of 4.802 (95% CI: 2.02 - 11.38) for participants who have breakfast after 10 am. The AOR is 3.98 (95% CI: 1.65 - 9.61), with a highly significant p-value of 0.001, reinforcing the strong association between late breakfast timing and skipping breakfast. Those who use snacks as a meal replacement have an unadjusted odds ratio of 3.27 (95% CI: 2.24 - 4.79), indicating a strong likelihood of skipping breakfast. The AOR is 2.90 (95% CI: 1.95 - 4.32), with a p-value of 0.001, highlighting a significant association. The unadjusted odds ratio for participants who spend pocket money on breakfast is 1.76 (95% CI: 1.20 - 2.58), suggesting that they are more likely to skip breakfast. A substantial p-value of 0.004 and an adjusted odds ratio of 1.25 (95% CI: 0.82 - 1.90) indicate that financial conduct has a significant influence on breakfast habits.

The results of the logistic regression analysis suggest that while gender and pocket money expenditures do not significantly correlate with skipping breakfast, the timing of breakfast and the usage of snacks as meal substitutes are significant predictors.

## Discussion

This study was conducted among 540 college students to determine their breakfast-skipping patterns and the factors influencing them.

Prevalence of breakfast skipping

The prevalence of breakfast skipping among college-going students from our study was 368 (68.2%). This result was consistent with a cross-sectional study done by Badrasawi et al. [19] in Palestine which reported that among 12-14 years of school-going children, 32% of them were found to have their breakfast daily. This indicates a higher prevalence in younger populations and it shows a concerning trend of skipping breakfast as they enter into higher education. Conversely, Moller et al. [20] from Australia reported that 45% of school-going students skipped their breakfast suggesting that skipping breakfast was a widespread issue across different age groups and educational contexts. Furthermore, Sincovich et al. [21] reported that among school-going adolescents 55.0% said they never skipped breakfast, 17.4% said they did so occasionally, 18.0% said they did it frequently, and 9.5% said they always skipped breakfast. This study emphasizes that a substantial proportion of school-going students engaged in skipping breakfast as compared with our study results.

A Saudia Arabian study done by AlTamimi et al. [22] reported that 52.8% of male adults aged 20 - 35 years had breakfast-skipping patterns. This study aligns with our study results as half of the proportion had their breakfast skipped among the adult population. Mansouri et al. [23] from Iran found that 4.9% of university students had breakfast less than one day per week. This result was in contrast to our results as our study population had a higher proportion of breakfast skipping patterns among college students. Khan et al. [8] from Bangladesh, Alshdifat et al. [10] from Jordan, Alhazmi [24] from Saudi Arabia, and Selvi et al. [9] from South India reported that 63.5%, 66%, 62.4%, and 66% of university students respectively missed their breakfast similar to our study. However, the varying proportion across the studies could be due to the study population and sample size characteristics.

Reasons for breakfast skipping

Our study found that nearly half of the participants were not found to have breakfast due to lack of time and one-fourth of participants do not like eating breakfast. Badrasawi et al. [19] reported that most of the school-going students wouldn’t feel hungry, half of them had no time for breakfast, and a small proportion reported with lack of time. Alshdifat et al. [10] research also stated that students lack time as they get up late which is the main reason for missing their breakfast. Selvi et al. [9] from Coimbatore found that lack of time, getting up late, non-availability of foods, not feeling hungry, and disliking food are the reasons stated by college students. These studies reported similar reasons stated by students as due to lack of time they have missed their breakfast. In the current scenario, college students become independent, they follow poor sleeping patterns which influences breakfast consumption.

Breakfast skipping and factors influencing breakfast consumption

Socio-demographic analysis in our study revealed significant associations between breakfast skipping and factors such as gender, socio-economic status, and year of study, with male participants, Class III socio-economic status, and second-year students more likely to skip breakfast.

Badrasawi et al. [19] reported a significant relationship between living in the village and skipping breakfast; however, gender was not a significant variable associated with skipping breakfast. Their study also elucidated that doing physical activity, shorter screen time, and sleeping before 10 pm influence breakfast consumption. Sincovich et al. [21] research reported that skipping breakfast was more common among women, seniors in school, and residents of rural, isolated, and socioeconomically challenged areas among children and adolescents, similar to our study results. The students from low socio-economic status tend to miss their breakfast more even if they reside with their families and hostels. Even though some researchers do not find any significant association with gender, our study described a significant male proportion who had a habit of skipping breakfast. Sincovich et al. [21] found that women were a significant proportion associated with breakfast skipping, which might be due to the difference between the study population.

AlTamimi et al. [22] found that the study participants with higher education levels had a significant association with skipping breakfast. This statement generally takes into account that higher education or people who graduated have breakfast-skipping patterns. In this study, the students from higher education tended to miss their breakfast even though it was highly vulnerable to health.

The study also reveals a significant correlation between meal timing and breakfast skipping, with 60 (80%) of individuals waiting over 12 hours between dinner and breakfast missing the meal, and 18 (94.7%) of those eating after 11 a.m. also skipping it. The body's natural hunger signals may be interfered with by this prolonged fasting phase, which could result in an erratic eating habit that encourages missing meals.

Additionally, frequent snacking and spending pocket money on breakfast are associated with higher rates of breakfast skipping, indicating important behavioral patterns. These findings suggest that eating habits and financial behaviors can influence meal patterns, particularly the critical morning meal. The act of consuming snacks between meals may help enhance feelings of fullness and reduce the likelihood of overeating during subsequent meals. This behavior could be reflective of an overall approach to eating that prioritizes convenience and immediate satisfaction over regular meal patterns [25].

Additionally, using pocket money for breakfast has wider implications for nutritional choices and personal accountability. Those who do occasionally set aside money for breakfast can do it after the fact, which could result in irregular meal intake. The cycle of skipping breakfast may be further reinforced by this discrepancy, highlighting the necessity of treatments that support consistent eating habits in people, especially in young populations.

Breakfast skipping and obesity

This study shows that BMI does not show a significant association with breakfast skipping. There was an inverse association found between obesity and breakfast consumption in the study by Mansouri et al. [23]. Nilsen et al. [26] from Spain also found that breakfast skipping was associated with obesity. According to data from cross-sectional and cohort studies, skipping breakfast raised the chance of being overweight or obese by 48% and 44%, respectively, according to a new systematic review and meta-analysis [27].

Strengths

This study shows the density of the problem at hand and the main factors contributing to it in the geographical area mentioned. Since the availability of the data in the region is limited, the study results are important to address the problem.

Limitations

This study was done among college students; therefore, the results cannot be applied to the general population. The confounding factors like patterns on lunch, in between snacking habits, and dinner were not emphasized on obesity. Further studies have to be done in the general population with various age groups, ethnicities, and professions.

Recommendations

Stakeholders can promote healthier eating habits that enhance academic achievement and general health by addressing these variables. Health education on the adverse effects of skipping breakfast on health has to be given to the general population, especially in schools and colleges. Availability of breakfast at schools, colleges, and work can improve eating habits.

## Conclusions

This study shows that a higher proportion of college students skip their breakfast at least once a week. This study also showed that gender, mealtime, and snacking as a meal substitute are important risk factors for college students who were skipping breakfast. These results underline the necessity of focused interventions to encourage better breakfast practices, stressing the significance of creating regular meal schedules and teaching students about the nutritional benefits of a well-balanced breakfast.

## Appendices

Questionnaire

A.    Personal Details

1.     Name of the participant:

2.     Age:

3.     Gender:

4.     Course:

a)     Medical

b)     Dental

c)     Nursing

d)     Engineering

5.     Year of Study:

a)     First year

b)     Second year

c)     Third year

d)     Fourth year

e)     Intern

6.     Residential status of the students:

a)     Hosteller

b)     With family

c)     With relatives

d)     With friends

7.     Per Capita Income (per month)

B.    Anthropometry

8.     Height (in cm) ____

9.     Weight (in kg)___

C.    Breakfast pattern:

10.  Which is the important meal? Breakfast/ lunch/ dinner

11.  Do you know the importance of having breakfast? Yes / No

12.   Do you know the importance of having balanced diet? Yes / No

13.  What do you prefer the most in your breakfast? Idly/ pongal/poori/tea/others_____

14.  What is your minimum duration between dinner and your next meal?

a)     6-8 hours

b)     8-10 hours

c)     10-12 hours

d)     More than 12 hours

15.  Timing of taking breakfast

a)     Before 8 am

b)     8-10 am

c)     10-11 am

d)     After 11 am

16.  How often do you miss breakfast in a week?

a)     1-2 days

b)     3-4 days

c)     5-6 days

d)     Everyday

e)     Never

17.  What is the reason behind skipping of breakfast?

a)     Getting up late

b)     Unavailability of food

c)     Don’t feel like taking anything in the morning

d)     Don’t like the taste

e)     To lose weight

f)      Others___________

18.  How do you compensate your missed breakfast?

a)     Tea/Coffee

b)     Biscuits

c)     Compensated in lunch

d)     I don’t compensate

19.  Consumption of snacks as meal replacement

a)     Once per week

b)     2-3 times per week

c)     At least 4 times per week

d)     All days per week

e)     Never

20.  Do you take any supplements to compensate with your breakfast? Yes/no

21.  How often do you spend your pocket money on breakfast? 

a)     Very often

b)     Sometimes

c)     Rarely

d)     Never

22.  Do you encourage others to skip breakfast? Yes/ No

D.    Health effects of skipping breakfast:

23.  Do you feel hungry most of the day by skipping breakfast? Yes/ no

24.  How often do you feel distracted in class due to skipping breakfast

a)     Very often 

b)     Sometimes

c)     Rarely

d)     Never

25.  Do you know the complications caused due to skipping breakfast? Yes/ no

26.  Have you noticed any weight gain? Yes/ no

27.  How often do you dizzy due to skipping breakfast ?

a)     Very often

b)     Sometimes

c)     Rarely

d)     Never

28.  Have you noticed gastric troubles like acidity while skipping breakfast? Yes/ no

29.  Are you willing to change the habit of skipping breakfast? Yes/no
